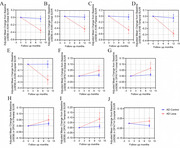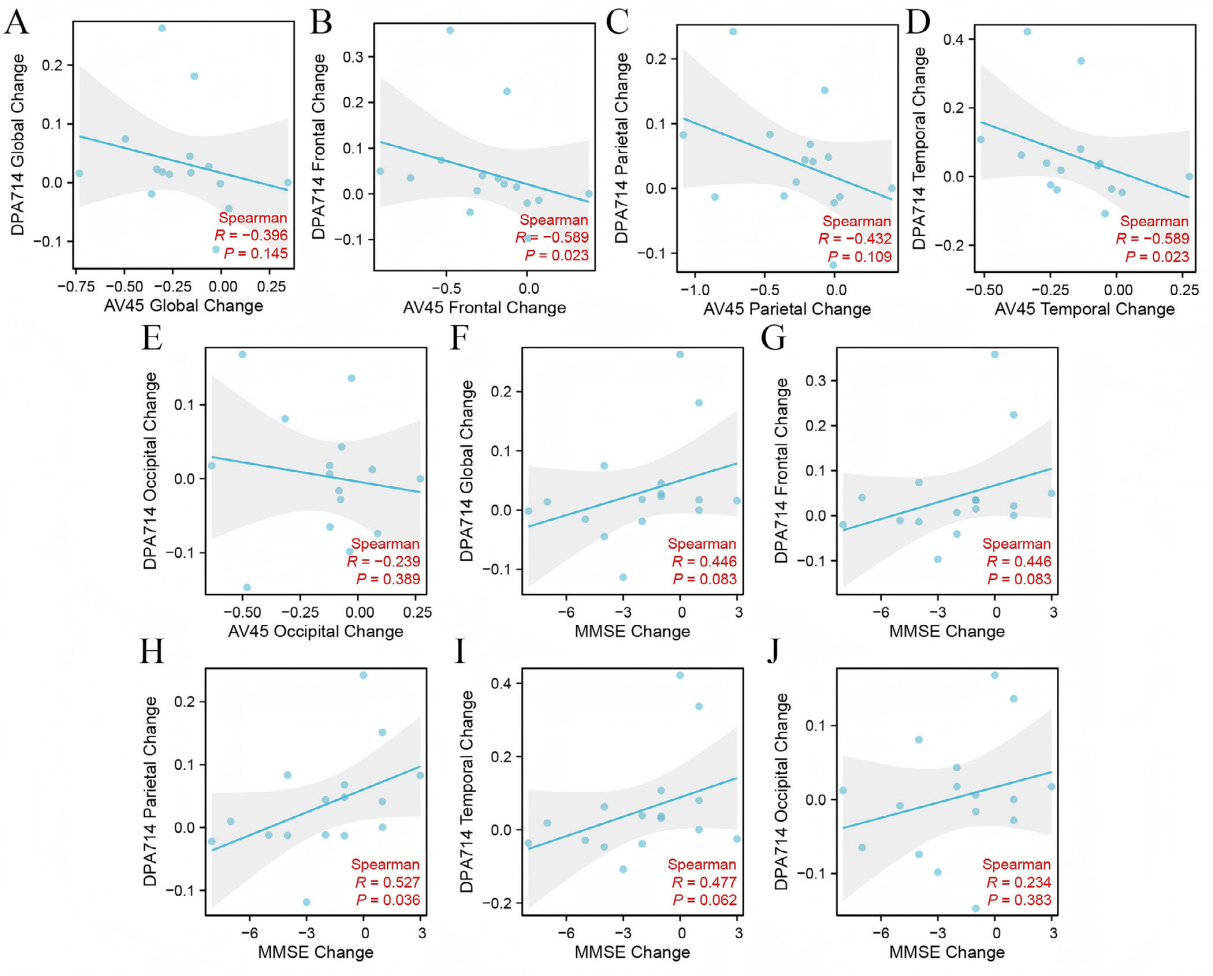# Microglial Activation Correlates with Amyloid Clearance and Cognitive Benefit in Lecanemab‐Treated Early AD Patients

**DOI:** 10.1002/alz70861_108511

**Published:** 2025-12-23

**Authors:** Xiao‐Hang Qian, Si‐Yue Chen, Pei‐Jing Cui, Miao Zhang, Hui‐Dong Tang

**Affiliations:** ^1^ Department of Geriatrics, Medical Center on Aging of Ruijin Hospital, Shanghai Jiao Tong University School of Medicine, Shanghai China; ^2^ Department of Nuclear Medicine, Ruijin Hospital, Shanghai Jiao Tong University School of Medicine, Shanghai China

## Abstract

**Background:**

Anti‐Amyloid β (Aβ) immunotherapy has been shown to clear Aβ and delay cognitive decline in Alzheimer’s disease (AD). Recent studies suggest that the efficacy of this treatment is linked to microglial activation. However, direct evidence from cohort studies is lacking. The 18‐kDa translocator protein (TSPO)‐positron emission tomography (PET) imaging allows for in vivo assessment of microglial activation.

**Method:**

The study included 10 early AD patients ((i.e., mild cognitive impairment or mild AD) treated with Lecanemab monoclonal antibody (10 mg/kg, biweekly) for 12 months, and 7 untreated early AD patients as controls. All participants underwent Aβ (AV45) and TSPO (DPA714) PET scans and cognitive tests at baseline and after 12 months.

**Result:**

No significant demographic differences were found between the Lecanemab and control groups. Both groups had similar proportions of APOE4‐positive individuals, cognitive levels, Aβ load, and microglial activation. At 12 months, the Lecanemab group showed a ‐0.34 SUVR for AV45/PET and a +0.05 SUVR for DPA714/PET. Correlation analysis revealed a positive correlation between increased frontal (r=‐0.59, *p* =0.023) and temporal (r=‐0.59, *p* =0.023) DPA714 SUVR levels and decreased AV45 SUVR levels. Additionally, an increase in parietal DPA714 SUVR was negatively correlated with MMSE score reduction (r=‐0.53, *p* =0.04).

**Conclusion:**

Microglial activation plays a crucial role in the efficacy of Anti‐Aβ immunotherapy, promoting Aβ clearance and delaying cognitive decline.